# Acceptability of PrEP among HIV negative Portuguese men who have sex with men that attended 2014 Lisbon pride fair

**DOI:** 10.7448/IAS.17.4.19734

**Published:** 2014-11-02

**Authors:** Luís Miguel Rocha, Maria José Campos, João Brito, Ricardo Fuertes, Jesus Rojas, Nuno Pinto, Luís Mendão, Julio Esteves

**Affiliations:** 1 GAT Portugal, CheckpointLX, Lisboa, Portugal; 2 GAT Portugal, Lisboa, Portugal

## Abstract

**Introduction:**

Consistent use of PrEP reduces HIV transmission from sexual practices amongst men who have sex with men (MSM) up to 92% [1]. Lisbon MSM cohort study estimates point that 59.3% of their participants at entrance (1593 HIV negative MSM enrolled between April 2011 and May 2013) were eligible for PrEP [2], according to the 2014 USA PrEP guidelines [3]. Studies about PrEP acceptability and implementation support policies aimed at increasing and rolling out its use. Hence, the exploratory study about PrEP acceptability in MSM at Lisbon.

**Materials and Methods:**

A street-based intercept survey, adapted from Mantell et al. study [4], was the one used on MSM attending the 2014 Lisbon pride fair. The survey included socio-demographic data, PrEP awareness and readiness to use it, probability of MSM's social network to also use it, promptness to join PrEP-related studies, type of PrEP warranted and condomless anal sex practice in the last six months.

**Results:**

A total of 110 HIV negative Portuguese MSM responded, with a median age of 33% and 84% of them identified themselves as gay. A majority of MSM were unaware of PrEP (59%); those that were aware, had heard of it trough CheckpointLx (31%), internet (22%) or health professionals (20%). 66% were likely or very likely to participate in PrEP-related studies. 57% of MSM were likely or very likely to use PrEP if available and reported that some, if not almost all of their social network, will do it too (70%). Type of PrEP preferred was oral, a pill a day (43%), followed by oral, intermittent intake (32%). Overall 41% of MSM had condomless anal sex practice in the last six months.

**Conclusions:**

In this MSM Portuguese sample, a general willingness to adopt PrEP was predominant, specially the oral daily intake. Forty-one percent of participants had had condomless anal sex practice in the last six months and therefore fitted within the criteria to be on Pre-Exposure Prophylaxis (PrEP), according to MSM Risk Index in 2014 USA PrEP guidelines. PrEP, when available in Portugal, should be a powerful tool for HIV prevention in this key population.
